# Fabp7 Is Required for Normal Sleep Suppression and Anxiety-Associated Phenotype following Single-Prolonged Stress in Mice

**DOI:** 10.3390/neuroglia3020005

**Published:** 2022-05-13

**Authors:** William M. Vanderheyden, Micah Lefton, Carlos C. Flores, Yuji Owada, Jason R. Gerstner

**Affiliations:** 1 Elson S. Floyd College of Medicine, Washington State University, Spokane, WA 99202, USA; 2 Department of Organ Anatomy, Graduate School of Medicine, Tohoku University, Seiryo-cho 2-1, Aobaku, Sendai 980-8575, Japan; 3 Steve Gleason Institute for Neuroscience, Washington State University, Spokane, WA 99202, USA

**Keywords:** fear, stress, lipid signaling, blbp, glia, anxiolytic

## Abstract

Humans with post-traumatic stress disorder (PTSD) exhibit sleep disturbances that include insomnia, nightmares, and enhanced daytime sleepiness. Sleep disturbances are considered a hallmark feature of PTSD; however, little is known about the cellular and molecular mechanisms regulating trauma-induced sleep disorders. Using a rodent model of PTSD called “Single Prolonged Stress” (SPS) we examined the requirement of the brain-type fatty acid binding protein Fabp7, an astrocyte expressed lipid-signaling molecule, in mediating trauma-induced sleep disturbances. We measured baseline sleep/wake parameters and then exposed *Fabp7* knock-out (KO) and wild-type (WT) C57BL/6N genetic background control animals to SPS. Sleep and wake measurements were obtained immediately following the initial trauma exposure of SPS, and again 7 days later. We found that active-phase (dark period) wakefulness was similar in KO and WT at baseline and immediately following SPS; however, it was significantly increased after 7 days. These effects were opposite in the inactive-phase (light period), where KOs exhibited increased wake in baseline and following SPS, but returned to WT levels after 7 days. To examine the effects of Fabp7 on unconditioned anxiety following trauma, we exposed KO and WT mice to the light–dark box test before and after SPS. Prior to SPS, KO and WT mice spent similar amounts of time in the lit compartment. Following SPS, KO mice spent significantly more time in the lit compartment compared to WT mice. These results demonstrate that mutations in an astrocyte-expressed gene (*Fabp7*) influence changes in stress-dependent sleep disturbances and associated anxiety behavior.

## 1. Introduction

Post-traumatic stress disorder (PTSD) develops rapidly, and is associated with long-term alterations in sleep and brain physiology. PTSD can elicit sleep abnormalities shortly after a traumatic event, with persistent changes in anxiety behavior. According to the American Psychiatric Association, sleep impairments are a diagnostic criterion for PTSD (2013). Poor sleep following trauma exposure is considered a predictor of subsequent PTSD severity [1–3]. However, very little is known regarding mechanisms that intersect sleep–wake processing and PTSD etiology. Therefore, understanding molecular and cellular events that interact between the regulation of sleep and symptomatology of PTSD will be important for targeting therapeutic strategies in treating this disorder.

This underscores the importance of the use of pre-clinical animal models that recapitulate hallmark traits of PTSD and associated changes in sleep [4]. Single prolonged stress (SPS) is a well-validated rodent model of PTSD where a subsequent 7-day isolation period is necessary for a PTSD-like phenotype to develop [5–10]. Previous studies showed that sleep alterations occur after SPS [11], and that sleep during this 7-day window following SPS exposure predict fear-associated memory impairments [12]. Further, optogenetic increases in sleep post-SPS exposure were associated with enhanced fear conditioning [13]. While these data indicate a relationship between sleep and the development of PTSD, specific molecules or cell-types that may integrate them, remain largely unexplored.

The astrocyte-enriched brain-type fatty acid binding protein, Fabp7, has been described to be associated with PTSD-like behaviors, as has the neuronal expressed Fabp3 [14]. For example, *Fabp7*-null mice exhibit enhancement of fear memory and anxiety [15]. We have previously shown that Fabp7 follows a circadian rhythm in gene expression broadly throughout mammalian brains [16,17], which is regulated by the core clock gene BMAL1 [18] and circadian transcriptional repressor REV-ERBα [19]. Fabp7 has also been shown to regulate sleep across phyla, including flies, mice, and humans [20–22]. Taken together, this suggest that Fabp7 may represent a functional node that regulates sleep and the etiology of PTSD.

In order to test this, we examined changes in sleep and anxiety behavior in *Fabp7* knock-out (KO) mice compared to C57BL/6N wild-type (WT) genetic background mice following SPS exposure. We observed a day-night reversal of sleep–wake phenotype following a full 7-day window post-SPS that is associated with cognitive disruption in a light–dark box anxiety test. These studies indicate that a genetic deletion of an astrocyte-enriched gene impairs sleep and cognitive processing, and provides a novel therapeutic target in lipid signaling pathways for treating PTSD.

## 2. Materials and Methods

### 2.1. Animals and Surgery

All animal procedures were carried out in accordance with the National Institutes of Health Guide for the Care and Use of Laboratory Animals, and approved by the WSU Institutional Animal Care and Use Committee (IACUC; ASAF# 6459 “Astrocyte Involvement in Stress Induced Sleep Alterations”).

Male, C57BL/6N (WT) and *Fabp7* KO mice (provided by Dr. Owada) were used for all experiments to eliminate the known impact of estrous cycle hormones on sleep and behavior [12,13,23]. Mice (60–90 days old) were housed in temperature (21–24 °C) and humidity-controlled (30–50%) rooms on a 12:12 light–dark cycle, and given ad-libitum access to food and water.

Mice destined for the EEG studies (*N* = 15) underwent one survival surgery to implant electroencephalographic (EEG) and electromyographic (EMG) recording electrodes (described below). Mice were given at least 10 days to recover from surgery prior to beginning the experiment. Animal well-being was assessed daily during the surgical recovery period. Any sign of illness or pain, including decreased motility and responsiveness, vocalizations, lack of appetite, decreased grooming, etc. was noted and treated in consultation with veterinary staff. Mice were housed singly following surgical procedures.

Survival stereotaxic surgery was performed on WT (*n* = 8) and *Fabp7* KO (*n* = 7) to implant the EEG/EMG sleep recording electrodes as previously published [13,21]. Aseptic surgeries were performed under isoflurane anesthesia. A midsagittal incision was made on the top of the skull, and the skin was retracted. After cleaning the surface of the skull, 4 holes were drilled through the cranium, and bare wire electrodes were inserted bilaterally over the frontal area and hippocampal area of the mouse brain for EEG recordings. Two flexible wire electrodes were threaded through the dorsal neck muscles for EMG recordings. All electrodes were connected to a six-pin connector which was attached to the skull via light-curable dental acrylic. Electronic connections were finalized through the six-pin connector to a Tucker Davis Technology (TDT) (Alachua, FL, USA) electrophysiology recording device.

### 2.2. Sleep Recording and Analysis

Following recovery from surgery, animals were housed individually and connected to the TDT recording system via a lightweight, flexible tether attached to a commutator (Sparkfun.com, Slip Ring) for free movement within the cage. The recording system was used to sample signals at 333 Hz, filtered between 0.1–100 Hz and amplified. Prior to analysis, signals were down sampled to 250 Hz. The four EEG electrodes were differentially referenced to obtain two independent EEG channels (frontal and hippocampal). Two EMG channels were also differentially referenced to obtain the EMG signal. Animals were given 48 h to acclimate to the tethers prior to beginning baseline recordings. During the acclimation period, animals were supplied food to last the duration of the EEG/EMG experimental recordings. While connected to tethers, animals were monitored daily for food, water, and health via visual inspection by a video monitoring system to avoid disturbing the animals.

Collected data were transferred from the recording PC, stored onto disk, and scored off-line in 10-s epochs to determine sleep/waking state using Sleep Sign software (version 3.3.6.1602) (Nagano, Japan). Three vigilance states were assigned: Wake, REM sleep and NREM sleep. Wake consists of visible EEG theta activity and high EMG activity; REM sleep consists of clear, sustained EEG theta activity and phasic muscle twitches on a background of low EMG; and NREM sleep consists of high amplitude, synchronized EEG and low EMG activity.

EEG and EMG signals were recorded for 24 h of baseline, after which the animals were unhooked from the recording system; single prolonged stress (SPS, described below) was performed. Following SPS, animals were reconnected to the recording system and seven subsequent days were recorded and scored. Data collected after SPS were compared to the baseline recording day using Graphpad Prism software. Sleep states were quantified as an average duration spent in state per hour (in seconds) over the light phase (ZT0–12) and dark phase (ZT12–0).

### 2.3. Single Prolonged Stress

**Figure 1.**
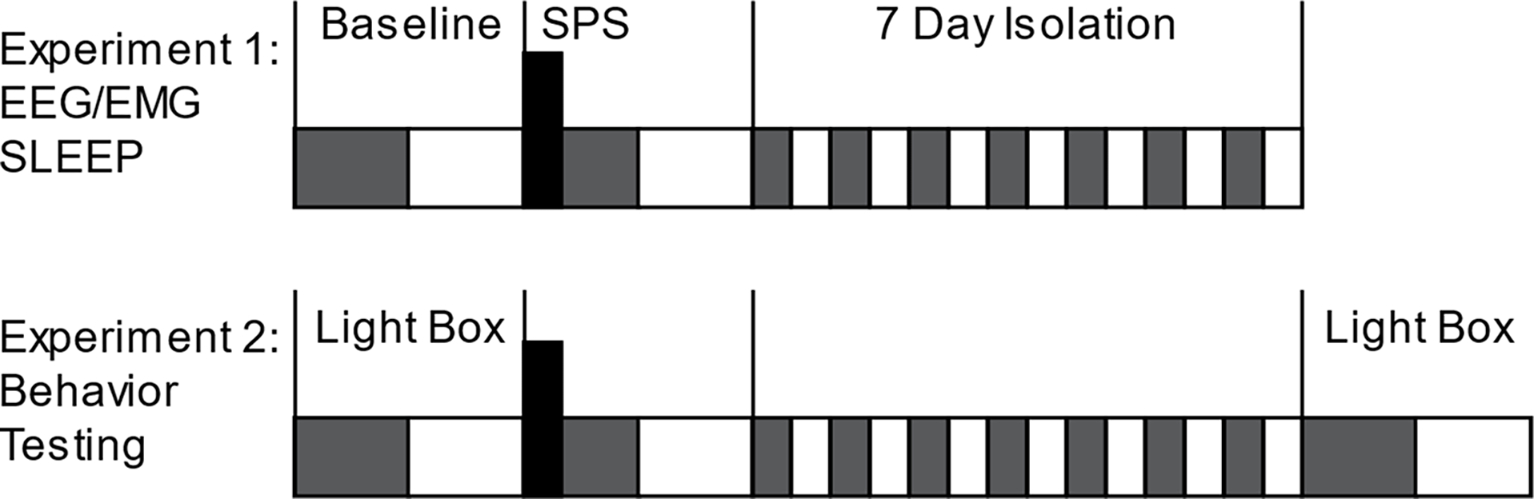
Experimental timeline. Animals were divided into 4 groups: WT/Control, WT/SPS, *Fabp7* KO/Control, and *Fabp7* KO/SPS. Experiment 1: Baseline EEG/EMG for sleep measures was recorded for 24 h in all groups, immediately followed by SPS exposure (Black box) at ZT12 for trauma exposed groups. Experiment 2: Separate groups of non-surgerized WT and *Fabp7* KO animals were tested using the light–dark box anxiety test (Light Box) at baseline and at 7 days after SPS (7 Day Isolation).

Single prolonged stress was performed similarly to previously published work [10,13,21]. Briefly, animals were exposed to 3 successive stressors at the start of the dark phase. First, physical restraint was performed for 2 h in custom-built plexiglass restraining devices. Next, the animals were placed in a (20 W × 20 D × 10 H cm) plastic bin containing 30 °C water and were forced to swim in groups of 6–8 for 20 min. Following a 15-min recuperation period in a towel-lined bin, the animals were exposed to 30 mL of ether vapors in a 2000 cc isolation chamber until fully anesthetized (<5 min). Ether exposure is a critical component in the development of the PTSD phenotype in rodents; substitution of an alternative anesthetic, such as isoflurane for ether, is insufficient to cause extinction retention deficits in fear-associated memory processing [23]. Afterward, the animals were returned to their EEG/EMG recording-cages where they were isolated for the following seven days (as shown in Figure 1).

### 2.4. Light–Dark Box Anxiety Testing

To assess baseline anxiety, animals underwent light–dark box testing. All animals, WT (*n* = 7), WT/SPS (*n* = 7), *Fabp7* KO (*n* = 8), and *Fabp7* KO/SPS (*n* = 8), were housed individually in the experimental room on a 12:12 light:dark schedule for seven days prior to beginning testing. SPS animals were then trauma exposed at ZT12 and allowed to rest for another seven days. The testing chamber consisted of two compartments, each measuring 7” W × 7” D × 12” H, separated by a 3” H × 3” W door with a metal bar floor (Coulbourn Instruments, Allentown, PA, USA). The right chamber was illuminated with white light and the left chamber was dark. The position of the animal was determined using infrared beams placed along the floor of the entire chamber. All testing was performed at ZT12. The animal was started in the illuminated compartment and allowed to freely explore both sides of the chamber for 300 s. Time spent on each of the light side and the dark side was measured, along with the number of transitions between compartments, and initial escape latency to the dark side. Supplementary methods can be found in Supplementary materials.

## 3. Results

### 3.1. Sleep–Wake Behavior

#### 3.1.1. SPS Changes in Wakefulness in *Fabp7* KO versus WT

**Figure 2.**
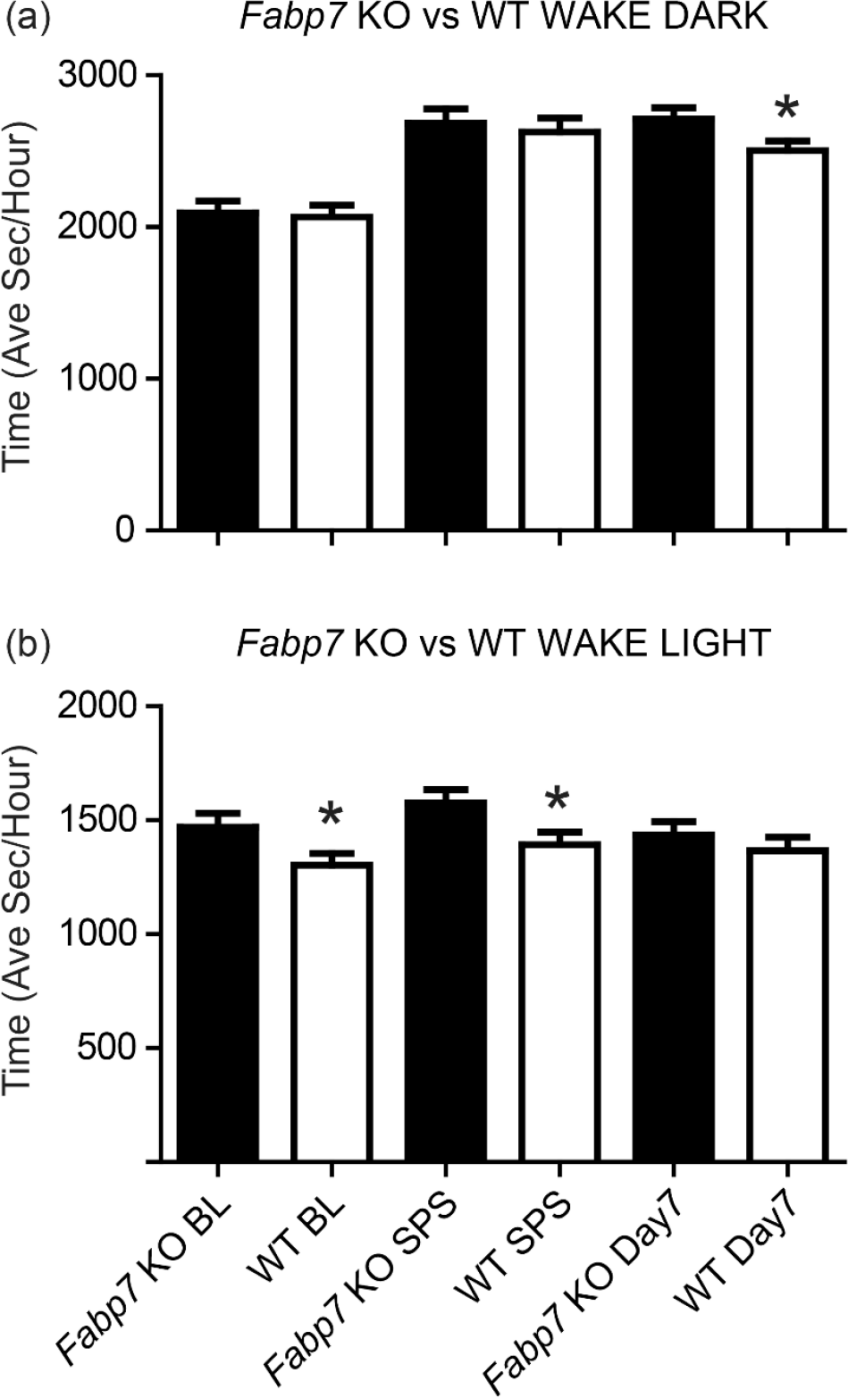
Differences in wake duration pre- and post-SPS in *Fabp7* KO versus WT mice. Wake duration was quantified during the (**a**) active-phase (dark period, ZT12–ZT24) and (**b**) inactive-phase (light period, ZT0–12) on baseline day (BL), the trauma exposure day (SPS), and after a 7-day window post-SPS (Day 7). Independent Student’s *t*-test revealed significant differences between *Fabp7* KO animals and WT controls, * = *p*-value < 0.05.

In order to determine whether Fabp7 plays a role in the interaction between sleep–wake behavior and PTSD-like phenotype, we measured sleep and wakefulness from EEG data of *Fabp7* KO and WT control mice prior to treatment (baseline), immediately following SPS, and after the 7-day window post-SPS. The experimental outline is shown in Figure 1. During the active-phase (dark period), we did not observe any differences in baseline wake time, or immediately following SPS (Figure 2a). However, after the 7-day window when SPS treatment is known to cause sleep-associated long-lasting effects on fear-associated behavior [12], we observed an increase in wake in *Fabp7* KO versus WT mice (Figure 2a). These effects were reciprocal in the inactive-phase. *Fabp7* KO mice had an increase in wake during the light phase in baseline and immediately following SPS treatment (Figure 2b). After the 7-day window post-SPS, there were no differences in wake time between *Fabp7* KO and WT mice (Figure 2b). These data suggest that the astrocyte-expressed Fabp7 impairs the normal sleep-wake responses to single-prolonged stress in mice in a time-of-day dependent manner.

#### 3.1.2. SPS Changes in Sleep Stages between *Fabp7* KO versus WT

While we observed a change in active-phase versus inactive-phase wakefulness between *Fabp7* KO and WT mice after the 7-day window post-SPS, we also were interested in determining whether there were specific changes in sleep staging based on genotype. During the active-phase (dark period), there were no differences in baseline amounts of non-rapid eye movement (NREM) or REM sleep (Table 1). However, we observed a decrease in NREM sleep after the 7-day window post-SPS (Table 1), that was associated with an increase in wake observed during the active phase, in *Fabp7* KO versus WT mice (Figure 2a).

During the inactive-phase (light period), we observed differences in REM sleep on baseline and immediately following SPS in *Fabp7* KO versus WT mice, but not after the 7-day window post-SPS (Table 2). Similarly, no differences between *Fabp7* KO and WT were observed during the inactive-phase for NREM during baseline or after the 7-day window post-SPS, just immediately following SPS (Table 2). Taken together, these data suggest that the effects of SPS on sleep suppression post-trauma are dependent on normal levels of Fabp7, and that Fabp7-associated differences in sleep may in turn influence post-trauma behavior.

### 3.2. Light–Dark Box Anxiety Test

**Figure 3.**
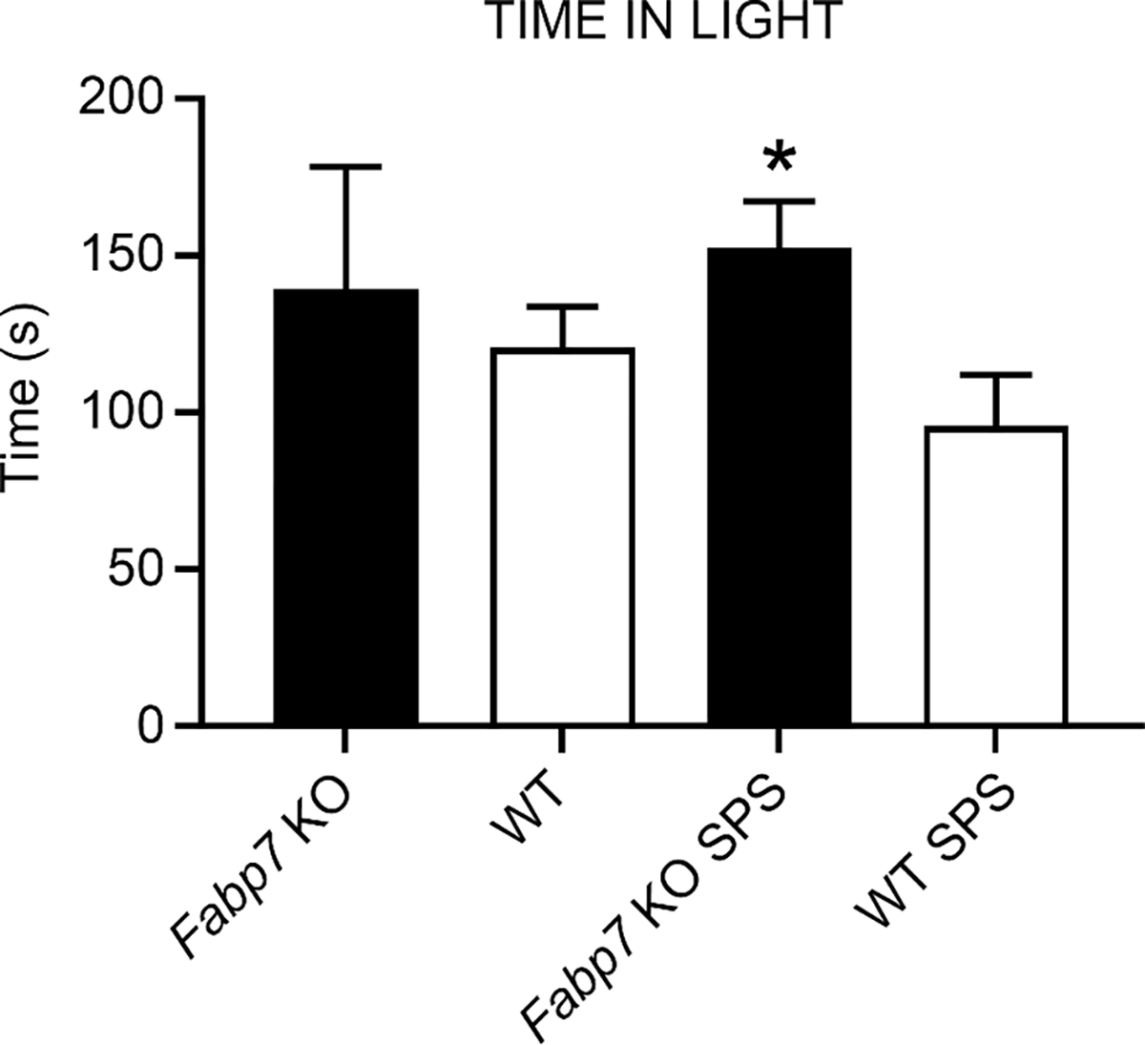
*Fabp7* KO SPS mice spend increased time in light compared to WT SPS mice. Time spent in the lit side of the light–dark box was measured while mice were able to freely explore the entire cage. One-way ANOVA showed statistical difference between the groups, with a Tukey multiple comparisons test showing that *Fabp7* KO SPS mice spent more time in the lit compartment than WT SPS mice, * = *p* value < 0.05.

The light–dark box anxiety test is an assay for determining anxiety behavior in rodents [24–27]. The light–dark box anxiety test is based on an unconditional aversion of rodents to brightly lit areas in response to mild stressors (e.g., novel environment and light). Here, we were interested in determining whether *Fabp7* KO mice show changes in innate anxiety following SPS treatment, compared to WT controls. We did not observe any baseline differences between *Fabp7* KO and WT mice in time spent in the lit compartment, however, *Fabp7* KO mice did have a significant increase in time spent in the lit compartment following SPS compared to WT controls (Figure 3). No differences were observed in the number of transitions (Appendix A) or in latency to the dark box (Appendix B). These data suggest that the normal anxiety response following SPS is dependent on Fabp7 expression.

## 4. Discussion and Conclusions

Clinical and animal studies have shown that fear-associated neuronal circuits are closed tied to the development and retention of PTSD [28,29]. In addition, sleep disturbances are tightly associated with PTSD, and may represent a point of intervention [30–32]. The neural circuits that have been implicated in PTSD include fear learning, emotional processing, arousal, and context processing circuitry [33,34]. However, much less is known about how glial cells, and in particular, astrocytes, may be playing a role in PTSD; however, the role of glia in sleep processing is beginning to be understood [22,35–37]. How non-neuronal cells such as astrocytes may be implicated in the relationship between sleep behavior and PTSD etiology remains poorly understood. Fabp7 is an astrocyte-expressed molecule that has been implicated in fear and anxiety-like behavior [15], cognitive processing [20,21,38] and sleep–wake regulation [20–22]. Here we show that Fabp7 is required for normal sleep suppression following trauma, using the SPS paradigm in mice. We also observed Fabp7-dependent disruption of anxiety-related phenotypes post-SPS.

Our studies show that the trauma-induced sleep–wake phenotype in *Fabp7* KO compared to WT mice changes between the active-phase and inactive-phase (Figure 1). Whether these differences can be partially accounted for by NREM and REM sleep stages over the light–dark cycle (Figure 2) will require future investigation. For example, optogenetic enhancement of sleep was shown to improve fear memory following SPS in rats [13]. Previously, we showed that SPS blocks sleep homeostasis; however, pre-trauma sleep deprivation did not exacerbate trauma-induced fear-associated memory impairments [23]. Here, we observed time-of-day-dependent differences in pre- and post-SPS sleep stages between *Fabp7* KO and WT mice (Tables 1 and 2). During the dark phase, we only saw differences in NREM sleep between *Fabp7* KO and WT mice following the 7-day window post-SPS (Day 7). However, during the light phase, differences in REM sleep were observed between *Fabp7* KO and WT mice during baseline and SPS, but these normalized by Day 7. The light phase post-trauma increases in REM sleep observed in *Fabp7* KO mice may be relevant for testing potential treatments for PTSD and cognitive function. The phosphodiesterase-4 (PDE4) inhibitor rolipram was shown to have anxiolytic effects in mice [39]. Rolipram treatment is known to rescue cognitive deficits following REM sleep deprivation for spatial working memory [40], or for contextual fear conditioning following total sleep deprivation [40,41]. Whether PDE4 represents a mechanistic pathway in neural or glial cells in the relationship between changes in sleep and cognitive processing [42] in our model will require more experimentation. In addition, future work in disrupting sleep or disrupting different stages of sleep (i.e., either NREM or REM) at various time-windows post-SPS will be needed to determine the role sleep plays in cognitive processing after trauma exposure.

Previous studies showed that *Fabp7* KO mice exhibit increased anxiety-like behavior [15]. Here, we observed that *Fabp7* KO mice spend approximately the same amount of time in the lit compartment as WT mice under baseline conditions, but spend significantly more time in the lit compartment following SPS. While this effect may appear to contradict findings by Owada et al. [15], it may mean that *Fabp7* KO mice have anxiolytic-like effects on cognitive processing following traumatic stress, or have an enhanced ‘freezing-like’ behavior when placed in the lit compartment. Therefore, future studies will be important to identify the precise mechanism that Fabp7 may be playing in astrocytes to affect sleep and anxiety following trauma in our model.

Astrocyte loss was shown following SPS exposure in the hippocampus of rats [43]. Chemogenetic technology targeting dorsal hippocampal astrocyte activation was also shown to be sufficient to attenuate stress-enhanced fear learning (SEFL), a PTSD-like behavior [44]. Using SEFL, hippocampal astrocyte expression of interleukin-1β (IL-1β) was shown to be increased [45]. SEFL exposure reduced immunoreactivity for the dorsal hippocampal postsynaptic density 95, PSD95, a synaptic maker, which was co-localized with astrocytes [44]. Previously, we showed that Fabp7 protein and mRNA oscillated in a circadian phase in the fine perisynaptic processes of astrocytes, and the mRNA is trafficked within the hippocampus based on time of day [46]. Fabp7 inhibition was previously shown to limit cytokine production and secretion of TNF-α and IL-1β [47,48], suggesting that a signaling cascade may exist between astrocyte Fabp7 activity and IL-1β post-trauma. The astrocyte Fibroblast Growth Factor 2 (FGF2), a mitogen that is involved in the signaling pathways for memory extinction and relapse [49,50], was shown to block PTSD behavior following SPS in rats [51]. Xia et al., also observed that intraperitoneal FGF2 administration inhibited SPS-induced hyperarousal and anxiety behavior [51]. Fabp7 transfection in U87 astrocytoma cells was shown to increase FGF2 expression [52], suggesting that FGF2 and Fabp7 may be linked in the development of PTSD behavior. Future studies determining the relationship of sleep and Fabp7 with other previously identified astrocyte factors that mediate PTSD-related phenotypes will be important for generating clinical therapeutic strategies for the treatment of PTSD.

## Supplementary Material

Supplementary

## Figures and Tables

**Table 1.** Active period sleep stage distribution. Non-rapid eye movement (NREM) and REM sleep time in minutes on baseline (BL), immediately following SPS, and after a 7-day window post-SPS (Day 7) in WT and *Fabp7* KO mice between ZT12-ZT24; Student’s *t*-test (*p*-value).

Dark Phase					

	WT		*Fabp7* KO		

NREM	Mean	SEM	Mean	SEM	*p*-Value

BL	1389.688	67.48	1349.896	69	0.680569
SPS	897.6042	82.37	830.1042	83.97	0.56674
Day 7	1021.042	56.28	813.2143	65.74	0.016713

**REM**					

BL	144.5833	14.5	156.25	13.59	0.557893
SPS	75.72917	12.12	85.3125	12.56	0.583581
Day 7	74.27083	8.361	73.80952	9.651	0.971073

**Table 2.** Inactive period sleep stage distribution. Non-rapid eye movement (NREM) and REM sleep time in minutes on baseline (BL), immediately following SPS, and after a 7-day window post-SPS (Day 7) in WT and *Fabp7* KO mice between ZT0-ZT12; Student’s *t*-test (*p*-value).

Light Phase					

	WT		*Fabp7* KO		

NREM	Mean	SEM	Mean	SEM	*p*-Value

BL	1991.25	45.7	1880.521	53.82	0.118468
SPS	1883.854	46.98	1747.708	49.05	0.046434
Day 7	1934.583	50.25	1873.214	49.07	0.386286

**REM**					

BL	306.5625	13.49	249.8958	12.77	0.002612
SPS	323.5417	12.89	275.8333	12.6	0.00879
Day 7	299.8958	14.65	291.5476	15.68	0.697698

**Figure A1.**
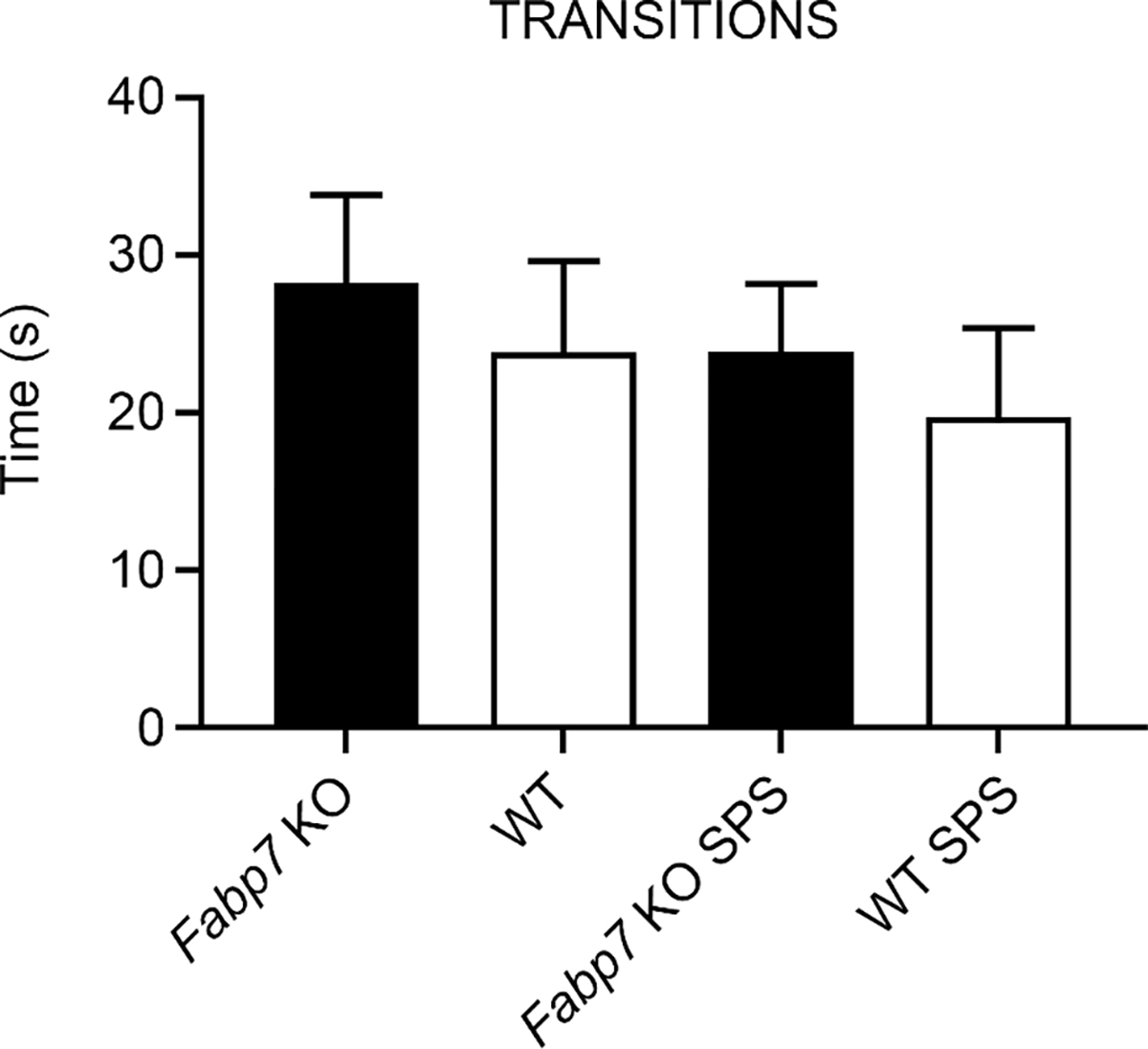
No differences in the number of transitions between WT and *Fabp7* KO in the Light–Dark box test. One-way ANOVA showed no statistical difference between the groups, pre- or post-SPS treatment.

**Figure A2.**
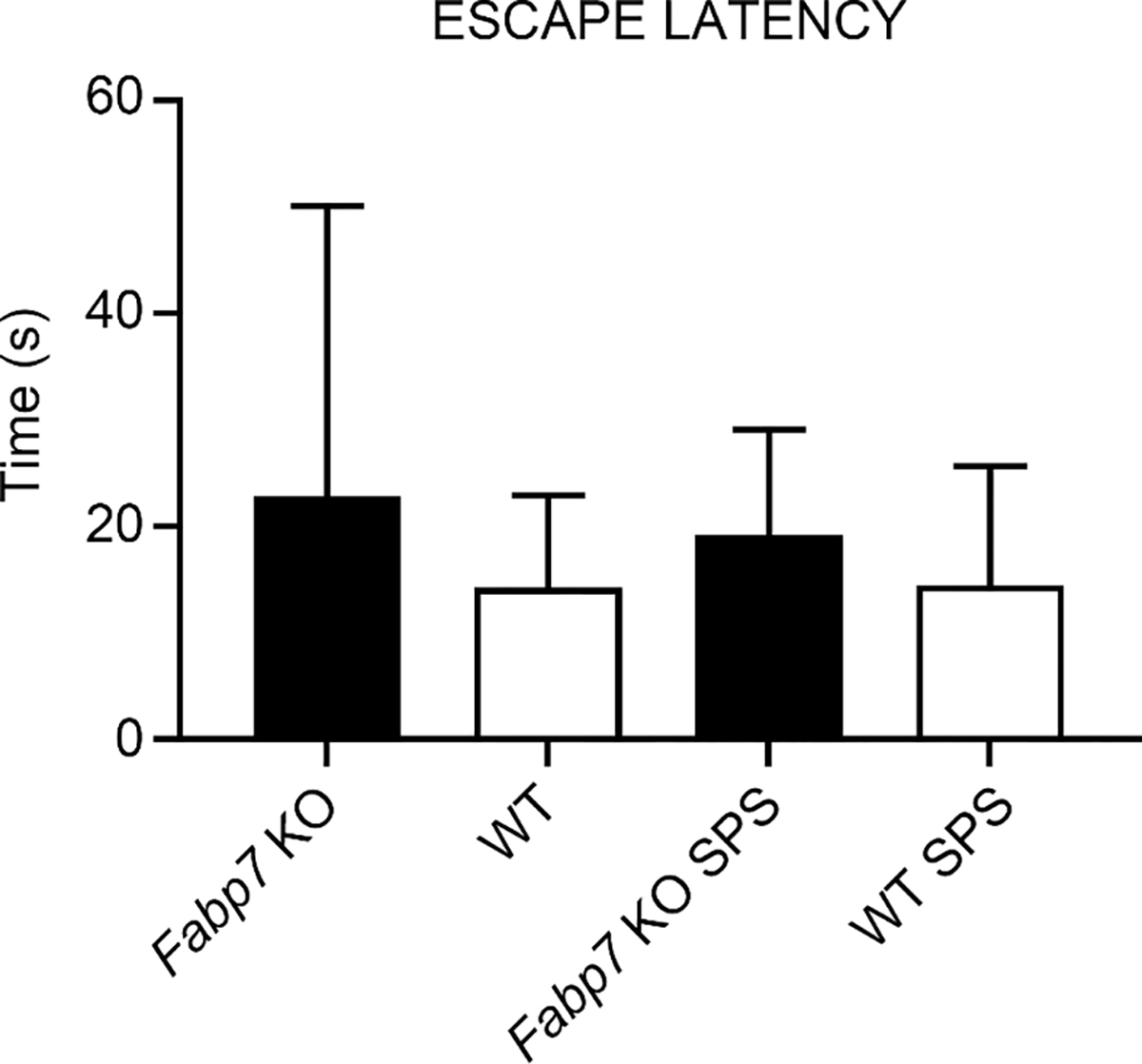
No differences in the escape latency between WT and *Fabp7* KO in the Light–Dark box test. One-way ANOVA showed no statistical difference between the groups, pre- or post-SPS treatment.

## Data Availability

Not applicable.
