# Supplementary material for: Fabp7 Is Required for Normal Sleep Suppression and Anxiety-Associated Phenotype following Single-Prolonged Stress in Mice
Source: Neuroglia. Author manuscript; Available in PMC 2023 Mar 10. (PMC10001429; doi:10.3390/neuroglia3020005)
Supplement: Supplementary [file NIHMS1846054-supplement-Supplementary.pdf]

Tethered electroencephalogram (EEG) and electromyogram (EMG)/microscopy in mice (duration of surgery > 1 hour).

Animals are anesthetized with either inhalation anesthetic (isoflurane 5% induction, 1-4% maintenance) or ketamine/xylazine cocktail. The fur is shaved or removed via depilatory cream from the top of the head. Animals are placed into a stereotaxic apparatus and body temperature is maintained using a heating pad or water blanket. The top of the head is then carefully scrubbed with Betadine using a new clean surgical sponge or sterile cotton swab. To scrub the site, we use a gradually enlarging circular pattern from the center of the proposed incision to the periphery. The sponge or swab is not brought back from the contaminated periphery to the clean central area. Next, we rinse the area that has been scrubbed using 70% alcohol. We repeat the scrub/rinse cycle for a total of 3 times, each time beginning at the center and proceeding to the periphery. At the onset of anesthesia, ophthalmic ointment is applied for corneal protection. Next, a dorsal midline incision is made, the temporalis muscle retracted, and the skull cauterized and thoroughly cleaned with a 3% hydrogen peroxide solution. The surface of the skull may be treated with a topical etchant and 3M bonding agent that improves bonding between bone and resin. Stainless steel screws (#000) or electrodes are placed into the skull and serve as electroencephalograph (EEG) electrodes. The screws are attached to teflon-coated stainless steel wires. Multi-stranded twisted stainless steel wire electrodes are sutured bilaterally in the neck muscles for recording of the electromyograph (EMG). EEG and EMG leads are attached to small connectors and affixed to the skull with dental acrylic and cyanoacrylate glue. In some cases, a sterile transparent chamber is inserted in one hemisphere intracranially. This procedure is similar to the placement of EEG screws described above. A physiological buffer solution designed for the CNS is used as a buffer to keep the brain moist during implantation. The chamber is closed to the environment but permits light capture for use with a camera objective. Incisions are closed with absorbable suture (4-0 or 5-0). At the end of the surgical procedure, buprenorphine is administered as a long-lasting analgesia. An antibiotic such as Baytril is administered. Animals are closely monitored following surgery until they are ambulatory. Subsequently, they are carefully observed daily (~5 mins/day).
